# Could Emergency Diseases Surveillance Systems Be Transitioned to Routine Surveillance Systems? A Proposed Transition Strategy for Early Warning, Alert, and Response Network

**DOI:** 10.3389/fmed.2022.670083

**Published:** 2022-03-28

**Authors:** Rana Jawad Asghar, Abdinasir Abubakar, Evans Buliva, Muhammad Tayyab, Sherein Elnossery

**Affiliations:** ^1^Global Health Strategists & Implementers, Islamabad, Pakistan; ^2^The WHO Regional Office for the Eastern Mediterranean (WHO EMRO), Cairo, Egypt

**Keywords:** early warning alert and response network, EWARN, humanitarian crisis, disease surveillance and control, World Health Organization, health emergency and disaster risk management

## Abstract

In humanitarian emergencies, traditional disease surveillance systems either do not exist to begin with or come under stress due to a huge influx of internal or external migrants. However, cramped camps with an unreliable supply of safe water and weak sanitation systems are the ideal setting for major disease outbreaks of all kinds. The Early Warning, Alert and Response Network (EWARN) has been supported by the WHO since the late 1990s to ensure health system capacity to identify and control risks early before they become major epidemics. These systems have been proven to be an excellent asset in reducing morbidity and mortality in humanitarian crises around the world. However, there is also a global challenge of transitioning them back to a regular or national monitoring system in their respective countries. This article is the result of in-country consultations arranged by the Eastern Mediterranean office of the World Health Organization. In these consultations, the unique local conditions and limitations of different countries were discussed to identify a way forward for transitioning these emergency disease surveillance systems into regular systems. After these discussions, different options were presented which could be further modified according to local needs. As there has not been any documented evidence of a successful transition of any emergency surveillance system, it is difficult to discuss or determine the gold standard for transition. As with any public health program being practiced in the field, local decision-making with some broad guidelines will be the best approach available. This article provides these guidelines and practical steps which could be further modified according to country needs.

## Introduction

Disease surveillance systems are understood differently by different people. For the vast majority, they are just systems that report numbers of diseased people in a population. The World Health organization describes disease surveillance systems as, “An ongoing, systematic collection, analysis and interpretation of health-related data essential to the planning, implementation, and evaluation of public health practice” (1). Where some components of the above definition are operational in varying countries at various strengths, the last three components are missing in most countries. Disease surveillance data is hardly ever used for any planning or resource allocation. With no relevance to decision-making processes, fewer resources are made available for surveillance.

Surveillance systems are of many types including population-based and sentinel surveillance systems. Population-based surveillance systems are theoretically more representative, but the data quality is normally poor. When high-quality data is required, sentinel systems are adopted in selected sites to ensure high-quality data capture. However, sentinel sites lack representativeness in most scenarios.

In humanitarian emergencies, the situation is even more complicated. There are huge population displacements and local health systems may already be strained. With cramped living conditions and a lack of safe water and sanitation, these populations are already vulnerable to various disease outbreaks. Existing structures of disease reporting may have been destroyed or under-reporting due to conflict or emergency or may just not be there to start with. It becomes even more critical to identify outbreaks quickly and contain them to protect the health of these displaced vulnerable populations. These humanitarian crises required the WHO to develop emergency surveillance systems that could be deployed quickly in the affected area. In this scenario, we normally deploy active disease surveillance systems to ensure any potential outbreaks are picked up well ahead of time to avoid a major epidemic. Over the years, these active systems have been modified according to the needs of specific countries.

Disease early warning systems have been supported by the WHO since the late 1990s. The concept was first introduced in 1999 when an “Early Warning, Alert and Response Network” (EWARN) was established in South Sudan (2). An “Early Warning, Alert and Response System” (EWARS) was also working in Darfur in the 2000s. The “Disease Early Warning System” (DEWS) was used for humanitarian crises, especially in Pakistan after the earthquake in 2005 and the 2010 historic floods (3). With some modifications according to host governments or implementing partner needs, there is different nomenclature for this type of disease surveillance system. For simplicity, we will be using EWARN as it is the most used name recently.

Multiple evaluations of WHO-supported EWARN have been completed. All of them are “timely surveillance systems that collect information on epidemic-prone diseases in order to trigger prompt public health interventions” (4). In Fiji, around 90% of those surveyed felt that the system detects outbreaks in a timely fashion and 77% agreed that they have had an impact on the health system of Fiji (5). In many countries, EWARN has filled the gaps of existing surveillance systems and may be operational for years after the end of the humanitarian crisis. In Nepal, an assessment by USAID found that their EWARN covered one-third of the districts in the country with timely weekly reporting (6). In the Americas, it was found to be highly sensitive in picking up outbreaks of Zika, Dengue, and Chikungunya (7). In Syria, an EWARN system was established in September 2012 and had a first review (8) in 2013 and most recently in 2018 (9). These evaluations demonstrate the expanding reach of EWARN even in challenging environments. In Somalia, EWARN has been credited with putting the brakes on epidemic and pandemic-prone diseases by US Centers for Disease Control and Prevention (10).

All of these early warning systems have proven to be an excellent asset in reducing morbidity and mortality in humanitarian crises around the world. However, there is also a global challenge of transitioning them back to a regular or national surveillance system in their respective countries. The WHO cannot be expected to continue indefinite material support for these EWARN systems after the humanitarian crisis is over, and once the WHO moves toward the transition or exit, there is a danger of loss of years of investments in a country's surveillance system. The WHO is clear on the continuation of EWARN beyond humanitarian crisis (11): “EWARN is an adjunct, not a substitute for the national disease surveillance system, and once the acute emergency phase is over, it should be re-integrated into the national surveillance system.”

However, transitioning and integrating into national disease surveillance systems has a varied level of success (12). There are many published guidelines and frameworks on how to make surveillance systems sustainable (13). Transitioning surveillance systems set up in humanitarian crises to regular surveillance systems is a big challenge. Timely data analysis could demonstrate a system's value to decision-makers by facilitating effective decision-making. This could in turn make the system a priority for resource allocations from decision-makers (14). That however is rarely documented. In a ten-year analysis of CDC-supported Influenza surveillance systems across multiple countries, donor exit or transition meant the country may scale down rather than improving operations (15).

The WHO recommends the concurrent elaboration of a clear exit strategy during EWARN implementation. However, because EWARN requires a quick implementation to deal with the destruction and chaos of emergencies, the full implication of “Exit discussions” is not necessarily well-understood by the host country. In places where the country has non-existent surveillance systems or those with only limited functionality, countries tend to rely on the WHO-supported system. Due to high data quality, its mandate is expanded both in the type of diseases and geographic areas (16, 17). However, it still stays outside the funding mechanism of governments. This creates a situation where when the emergency nature of the surveillance system is long past, the countries are then reluctant to transition to regular surveillance systems. That reluctance can be due to many reasons. More than financing challenges it can be the fear of losing a good quality disease surveillance system that they cannot implement on their own.

The sustainability of surveillance systems has always been a challenge, especially those surveillance systems initially set up and supported by international donors. To ensure that there is proper ownership from the host country (which in turn will help in transition) Moore et al. mention certain important factors for the sustainability of a surveillance system (13). These factors range from both donor and host country having the same agenda to local level input and utilization of data.

Exit strategies are a challenge for any donor-supported program. Normally exits occur in three different ways (18).

1. Phasing down
2. Phasing out
3. Phasing over

Phasing down means to curtail the activities, staff, and geographic area in view of saving resources. Phasing out is to exit without any long-term sustainability of the program. Phasing over is to properly phase the program to a local partner. Unfortunately, we see the first two outcomes happen more than the third.

UNESCO notes a few key challenges to the successful exit or transition strategy of any donor-supported activity such as a lack of shared understanding or meaning and relevance of an exit, and limited preparation for it (19). Although there is a lot of published work on successful implementation and evaluation of EWARN in multiple countries, there is scant evidence on the outcome of transition or exit (2). Lack of coordination and buy-in from all stakeholders destroy the sustainability of donor-supported disease surveillance systems (20). In a multicountry study on the transition of donor-supported countries, multiple challenges were identified for the successful transition of a program, and all challenges except one were attributed to the host country (21). As there are no published case studies of successfully transitioning an emergency surveillance system to a routine disease surveillance system in a host country, the Eastern Mediterranean Office of the World Health Organization arranged multiple series of consultations among regional countries to identify challenges and a way forward.

This article is based on the results of these regional consultations.

## Discussion

A 100 years on since the Spanish Influenza pandemic, humankind has once again faced a pandemic of unprecedented scale. COVID-19 has shown how critical a good disease surveillance system is not only for the health of the population but also for national economies and security. The pandemic has also pushed countries together with the WHO to strengthen their surveillance systems. This may be the first time that we observe a proper transition of early warning surveillance systems to regular surveillance systems. There is not a one size fits all approach to transition for different countries. The approach needs to be modified according to specific country resources, existing structures, and need assessments.

COVID-19 has shown how critical it is to keep our disease early warning surveillance and outbreak response systems fully functional. The cost of keeping these systems is much lower than the expensive lockdowns which many countries implemented. A devastating pandemic is a serious blow to the health and economy of the whole world but there is also a silver lining. There will be more interest both by host governments and international partners in keeping these disease early warning systems fully functional. That being said, we need to use this opportunity to build long-lasting sustainable systems to ensure we are not surprised by the next pandemic!

Not many donor-supported programs are sustained after the departure of resources. One reason for gross lack of sustainability is a lack of incentive for all in program results (22). From the start of the program, local stakeholders and government should be aware of their needs and ensure that it addresses and incorporates them in its own objectives. This will increase ownership and the chance for sustainability after the end of external resources.

**Here are some key Scenarios:**

1. The country has no desire to incorporate EWARN after the end of a crisis
  This could be due to many reasons such as a weak economic situation in the country and/or a health sector that is not properly funded. In this instance, the health system does not have enough resources earmarked for surveillance and the country does not realize how the data could be used to facilitate effective resource utilization.
2. The country has a desire to incorporate EWARN
  The country may appreciate the value of surveillance systems including EWARN but may already have many other existing donors supported vertical surveillance programs. In this case, there are multiple donors supporting different vertical disease surveillance systems. Donors are usually very protective of their resources, wanting them to be used only for *their* specific objectives. Even when in-country leaders of these programs have a desire to incorporate components of EWARN, their headquarters are unable to agree with them due to legal, programmatic, financial, or political reasons.
3. The country wants to keep EWARN as a national surveillance system
  Even when country leadership shows a desire to keep EWARN as their national surveillance system, there can still be challenges such as not realizing that active disease surveillance systems (EWARN) are more resource-intensive than routine national surveillance systems. Secondly, unless a country has dedicated resources to run a surveillance system it will be difficult to operate or expand EWARN after the end of donor support.

## Recommendations

Exit without a transition should never be a strategy. In the worst-case scenario, some components of EWARN must be sustained by the country. Incorporation of the data needs of health departments beyond EWARN should be facilitated as soon as feasible. This strategy may increase the workload of EWARN and be criticized by some donors, but it will help to cultivate value for subsequent maintenance of the surveillance system beyond the current humanitarian crisis. Actionable information beyond EWARN helps Departments of Health to demonstrate their efficiency outside donor-supported areas. Thinking about how EWARN could be used in non-traditional ways that align with in-country values will help encourage ownership. The more useful it becomes before the transition or exit date, the more interest there will be from the government to own the program.

Below we outline some important steps that could help smooth the transition of EWARN, but each will need of course to be tailored to each country's specific needs. Different countries have different styles of government and unique legislative and financial regulations. Understanding these complexities early on will help facilitate a smooth transition for the program.

### Discussion With Government

Early in the process start discussions with the respective government and explain that EWARN is a temporary system that will not replace current or non-existent disease surveillance systems. This message needs to be conveyed and discussed at all levels of government. The government needs to be requested to deputize professionals who have background expertise in surveillance to be posted within the EWARN structure. A steering committee needs to be established (if not already present) with Government leadership and should include all key stakeholders (both government and non-government). This committee should meet at least once every 6 months, and every quarter during the year of transition. The Ministry of Finance/Planning should be contacted to be part of the steering committee. Advocacy meetings and seminars should be arranged to show with precise numbers how much mortality and morbidity were prevented by the surveillance system.

### Broader Stakeholder Meetings

Most donor organizations are very good at holding stakeholder meetings with the host country but most of “these meetings” are used as methods of one-sided communication. Donors must direct attention to mapping the stakeholders and identifying the appropriate level of participation (decision-makers and technical teams). It is also very important to ensure proper participation in these meetings and listen to the suggestions and ideas from stakeholders and identify opportunities for wider ownership of the surveillance system by sharing information and responsibilities. Providing meaningful participation and use of information to a wider group of stakeholders will help to transition EWARN to existing surveillance systems. It is also helpful to document the minutes of all meetings in a timely fashion and share them with all stakeholders.

### Evaluation of Surveillance System

Surveillance systems should be periodically evaluated using standard evaluation tools. Surveillance evaluation should not be limited to just EWARN, but candidate surveillance systems should also be evaluated for the possible transition of full or partial components of EWARN. Evaluation should be done with the full partnership of local stakeholders representing both the government and private sector. Results of the evaluation should be shared and discussed with all stakeholders in a timely manner.

### Discussions on Post Transition/Exit Shape of EWARN

One of the greatest challenges to the sustainability and smooth transition of the externally funded programs is that after the end of support, donors insist on keeping the program objectives intact. Keeping the original objectives operational is too costly for most host countries. Unfortunately, it then becomes a zero-sum game situation. To avoid this pitfall, we must consider the following issues: Does the country still need a fully dedicated active surveillance system after the close of the humanitarian crisis? What components of EWARN will the country need in long run? Is there a good existing surveillance system where disease early warning components could be housed? How do we identify and protect the optimal utilization of human resources and best practices developed and improved by host countries during the implementation of EWARN?

### Preparing a Road Map of Transition

Many times, roadmaps are prepared outside the country or in donor offices and then approval is sought from the government. The major reason many transitions fail is that though they are “Government approved,” there is no real government input. The worst part is that a local process is initiated but their recommendations are ignored by the donor who has their own timeline and processes. To be a successful roadmap we need to do the following: the most senior government official available with decision authority on both management and financial matters should chair the committee. All key identified stakeholders need to sit down and come up with a clear objective (transition or exit). An agreed timeline with doable targets will help everyone to be on track. The timeline should have clarity on the role and responsibilities required to reach each milestone. Monitoring meetings need to happen on a regular basis to see progress on each milestone and flag any issues.

### Mechanisms of the WHO Long-Term Relationship With the Country

After the transition, the WHO should provide technical support where needed to ensure a viable disease surveillance system for the country and global health security for all. Some mechanism of remote technical assistance should be established to provide the required input by experts. The local WHO office surveillance lead should provide high-level technical input if required. After the transition, some field visits by the WHO could advocate the government for continued investment in the surveillance system(s). Plans to conduct surveillance training activities for local staff will help to keep the focus of in-country decision-makers on the importance of disease early warning systems. A WHO-supported cost-benefit study could also strengthen Ministry of Health arguments for more resource allocation in a given country. Joint government and donor committees on disease surveillance could ensure that surveillance is coordinated in the country in an effective manner, to be used for the *actual* benefit of the population.

### Limitations

This article is the result of in-country consultations arranged by the World Health Organization which included countries with existing EWARN or similar disease surveillance systems. In these consultations' different countries' unique local conditions and limitations were discussed. After these discussions, different options were presented which could be further modified according to local needs. As there has not been any documented evidence of a successful transition of any emergency surveillance system, it is difficult to discuss or determine the gold standard for transition. As with any public health program being practiced in the field, local decision-making with some of these broad guidelines will be the best approach available.

## Author Contributions

All authors listed have made a substantial, direct, and intellectual contribution to the work and approved it for publication.

## Conflict of Interest

The authors declare that the research was conducted in the absence of any commercial or financial relationships that could be construed as a potential conflict of interest.

## Publisher's Note

All claims expressed in this article are solely those of the authors and do not necessarily represent those of their affiliated organizations, or those of the publisher, the editors and the reviewers. Any product that may be evaluated in this article, or claim that may be made by its manufacturer, is not guaranteed or endorsed by the publisher.
